# Prevention of type 2 diabetes in migrant populations from low- and middle-income countries living in high-income countries

**DOI:** 10.1007/s00125-025-06465-9

**Published:** 2025-06-07

**Authors:** Louise Bennet, Charles Agyemang

**Affiliations:** 1 Department of Clinical Sciences, Lund University, Malmö, Sweden; 2 Department of Public and Occupational Health, Amsterdam Public Health Research Institute, Amsterdam UMC, University of Amsterdam, Amsterdam, the Netherlands; 3 Division of Endocrinology, Diabetes, and Metabolism, Department of Medicine, Johns Hopkins University, Baltimore, MD USA

**Keywords:** Culturally sensitive interventions, Diabetes complications, Equity, diversity and inclusion, Implementation, Low- and middle-income countries, Migration, Mortality, Prevention, Review, Social determinants of health, Type 2 diabetes

## Abstract

The rising prevalence of type 2 diabetes among migrant populations from low- and middle-income countries (LMICs) living in high-income countries (HICs) presents a significant public health challenge. Migrants often experience lifestyle changes, socioeconomic disadvantages and barriers to accessing healthcare, which can increase their risk for type 2 diabetes. This review explores the prevention and management of type 2 diabetes in these migrant populations, emphasising the need for culturally sensitive strategies. Key interventions for mitigating the burden of type 2 diabetes include tailored dietary and physical activity programmes, community-based initiatives and healthcare models that consider social determinants of health and integrate traditional beliefs with evidence-based practices across genders. Addressing language barriers, improving health literacy and engaging trusted community leaders are all critical for effective intervention uptake. Additionally, policy measures to reduce structural inequalities, such as improving healthcare access and food security, are essential. Future research should focus on evaluating the long-term effectiveness of culturally sensitive interventions across gender and different migrant populations and on scaling successful models for broader implementation.

**Graphical Abstract:**

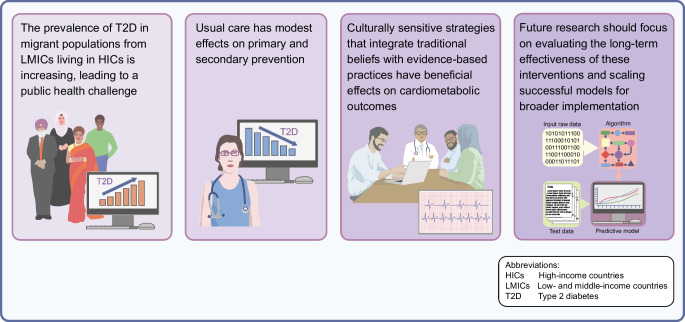

**Supplementary Information:**

The online version contains peer-reviewed but unedited supplementary material including a slideset of the figures for download, available at 10.1007/s00125-025-06465-9.

## Introduction

Type 2 diabetes is a rapidly growing global health challenge that is associated with significant morbidity and mortality and a substantial economic burden [1]. Three in four adults with type 2 diabetes live in low- and middle-income countries (LMICs) [1], and migrant populations from LMICs residing in high-income countries (HICs) are particularly affected by type 2 diabetes and related complications compared with host populations [2]. However, data on the effectiveness of intervention strategies for the prevention and management of type 2 diabetes among migrant populations are limited. This review explores the prevention and management of type 2 diabetes among migrant populations from LMICs living in HICs, and highlights the need for effective strategies to mitigate the growing burden of type 2 diabetes in these vulnerable populations. In this review the terms ‘ethnicity’ rather than ‘race’ or ‘ancestry’ and ‘gender’ rather than ‘sex’ are used to reflect the culture of migrant populations’ geographical origin, for example customs, language, history and religion [3].

## Migration patterns from LMICs to HICs

**Fig. 1:**
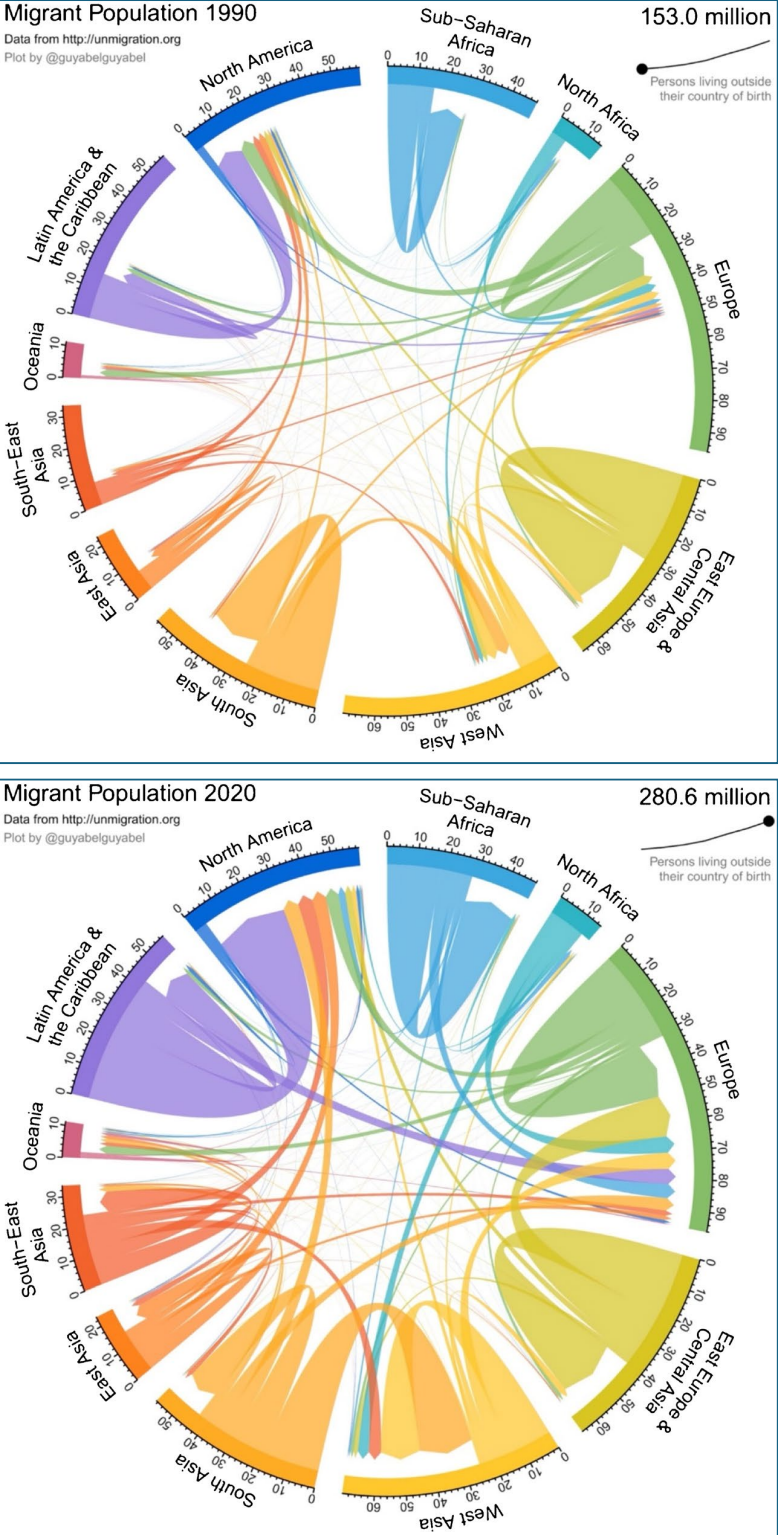
The global flow of migration in 1990 and 2020. The chords in the diagrams represent the connections between the places of birth (at the base of the chords) and places of residence (at the arrow heads of the chords). The width of the base of a chord corresponds to the size of the migrant population in millions. Chords are ordered relative to their size, with the largest migrant populations plotted at the beginning of the region segments. Reproduced from Abel [116] with permission. This figure is available as part of a downloadable slideset

The global geopolitical rivalry between the USA and the Soviet Union, and their respective allies during the Cold War, has resulted in millions of refugees from Vietnam, Korea and subsequently Afghanistan and Iraq, and more recently Syria, Ukraine and Venezuela [4]. Globally, the greatest increases in numbers of international migrants from 1990 to 2020 were seen in Europe (from 49.6 to 86.7 million international migrants), Asia (48.2 to 85.6 million international migrants) and North America (27.6 to 58.7 million international migrants) [5] (Fig. 1, electronic supplementary material [ESM] Fig. 1). This unstable global situation continues and the global political risk at the start of 2023 was at a 5 year high [6].

These recipient regions have received a large proportion of migrants from LMICs resulting in diverse populations. For example, in 2022 more than 20% of the populations of Luxembourg, Malta, Cyprus, Austria and Sweden were born abroad, mostly outside the EU [7]. A large number of migrant populations from LMICs to HICs are, to a high degree, refugees or family reunion migrants who often end up in vulnerable positions and face poorer health outcomes than the host populations [8].

Besides political instability, another future driver of migration is global warming, with 2024 being the warmest year since global records began in 1850 [9]. Half of the world’s population lives in countries rated as being at high or extreme risk for climate vulnerability. Climate change will force people to migrate, especially those living in sub-Saharan Africa, East Asia and the Pacific and South Asia regions, which together represent 55% of the population of LMICs [9]. Type 2 diabetes prevalence is high in these regions and climate change makes people living there more vulnerable to heat stress and dehydration [10]. It is estimated that, without climate and development action, by 2050 climate change will force 143 million people, or 2.8% of the population of these regions, to migrate to cooler regions due to climate-related health problems [11].

## Type 2 diabetes burden in migrant populations

A meta-analysis of type 2 diabetes prevalence among migrant groups in Europe showed that migrants from South Asia had the highest prevalence, with a nearly fourfold higher pooled OR compared with European populations, followed by those from the Middle East and North Africa, sub-Saharan Africa and South and Central America [12]. The odds of type 2 diabetes were over sixfold higher in migrants from Bangladesh, over fivefold higher in Pakistani migrants and fourfold higher in Indian migrants than in European populations [12]. The meta-analysis also showed that the magnitude of the ethnic differences in type 2 diabetes prevalence was greater in migrant women than in migrant men, relative to the host European populations [12]. A higher prevalence of type 2 diabetes has also been reported among migrants from LMICs living in other HICs compared with the host populations, including Australia, Canada and the USA [13–21]. In an Australian study, the odds of type 2 diabetes were over six- and sevenfold higher for men and women, respectively, born in the Pacific Islands and over fivefold higher for men and women born in Southern and Central Asia than in the Australian-born reference population [13].

Migrants from LMICs living in HICs also appear to develop type 2 diabetes 10–20 years earlier than European populations [22, 23], even at lower BMI levels [17, 24, 25]. The Healthy Life In an Urban Setting (HELIUS) study found a 20 year earlier onset of type 2 diabetes among South Asian Surinamese, African Surinamese, Ghanaian, Turkish and Moroccan migrants than among European Dutch participants [22]. A nationwide Swedish study on type 2 diabetes incidence reported a mean age at diagnosis of approximately 50 years in first-generation migrants from LMICs and 43.6 years in second-generation migrants, that is, approximately 10 and 16 years earlier than in people with type 2 diabetes without a migration background [23]. Furthermore, the prevalence of prediabetes (i.e. impaired fasting glucose or insulin resistance) is reported to be higher in migrants from LMICs than in the host populations, [19, 26, 27], predisposing migrant populations to a high risk of developing type 2 diabetes.

Regarding complications of type 2 diabetes, a 20-year follow-up of the UK-based Southall And Brent Revisited (SABRE) study found that the risk of stroke was almost twice as high in South Asian individuals with diabetes and over twofold higher in African Caribbean individuals with diabetes than in their European counterparts [28]. Similarly, the risk of microvascular and macrovascular complications of type 2 diabetes is reported to be higher in migrants than in their host European populations, although the magnitude of the risk varies across migrant populations [29–31]. In the All New Diabetics in Scania (ANDIS) cohort study, which included over 12,000 people diagnosed with diabetes in southern Sweden and followed for a decade from 2008, those born in the Middle East presented with a more insulin-deficient phenotype and genotype than Swedish-born participants and a higher risk of coronary events but lower risk of chronic kidney disease [32]. Paradoxically, data from the Swedish National Diabetes Registry showed lower all-cause and cause-specific mortality in first-generation migrants from LMICs with type 2 diabetes than in Swedish-born participants with type 2 diabetes [23].

Nevertheless, type 2 diabetes contributes to increased CVD risk and, therefore, considering the high type 2 diabetes burden and earlier onset in migrants from LMICs, primary prevention should be prioritised. Understanding the burden of type 2 diabetes in migrant populations from LMICs is crucial for developing targeted public health interventions, improving healthcare delivery and reducing health disparities.

## Potential drivers for type 2 diabetes in migrants from LMICs living in HICs

**Fig. 2:**
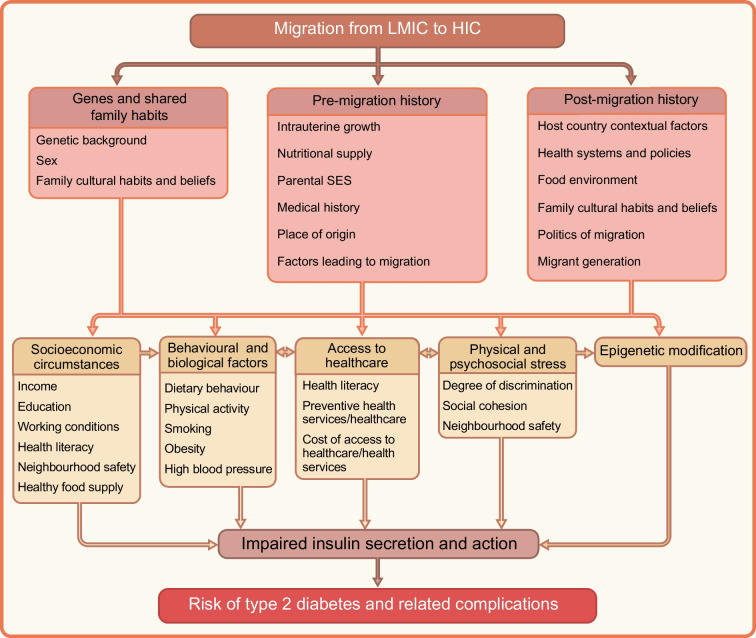
Potential drivers for type 2 diabetes in migrants from LMICs living in HICs. Drivers are related to genes and shared family habits, pre-migration history and post-migration history. Adapted from Agyemang et al [2] under the terms of the CC BY 4.0 Attribution License (http://creativecommons.org/licenses/by/4.0/). This figure is available as part of a downloadable slideset

Although migrant groups from LMICs have a relatively high prevalence of type 2 diabetes and related complications compared with the host populations in the destination HICs, the key underlying causes are still not well understood. It is recognised, however, that the causes are multifaceted, including pre‐migration factors (e.g. intrauterine growth, nutritional supply, parental socioeconomic status (SES), health history, place of origin), genetic predisposition and shared family habits, and post-migration factors (e.g. acculturation-related lifestyle changes, cultural traditions, physical and psychosocial stress, socioeconomic circumstances and inequalities, barriers to healthcare access and epigenetic modifications) [2] (Fig. 2).

### Pre-migration factors

Evidence indicates that adverse early life factors such as low birthweight and malnutrition during childhood have a profound impact on cardiometabolic disease outcomes such as type 2 diabetes in adulthood [33]. In a study conducted among African migrants in Europe, markers of poor childhood SES and early life nutritional status were found to be associated with abdominal obesity in men and women and with type 2 diabetes among men [34]. Many migrants, especially those originating from LMICs, have been exposed to a poor nutritional environment during early life because of poor socioeconomic circumstances, which may expose them to a high risk of type 2 diabetes on migration to food-abundant environments in HICs [35]. This phenomenon has been linked to the thrifty phenotype hypothesis, which postulates that people exposed to poor early life circumstances develop an adaptive ‘thrifty’ phenotype that increases survival under these circumstances. However, this phenotype becomes unfavourable in individuals who transition to food-abundant and sedentary environments, which are commonly seen in HICs [36].

### Post-migration factors

Post-migration factors such as changes in lifestyle relating to dietary behaviour, decreased physical activity levels, increased psychosocial stress and limited access to preventive services and curative healthcare can all have a profound impact on the development of type 2 diabetes among migrants [2]. Moreover, cultural changes following migration may influence migrants’ lifestyle and behaviour and subsequent risk of type 2 diabetes. 

Acculturation, that is, the process by which individuals are assimilated into a culture that is not their native culture, plays an important role in shaping type 2 diabetes risk perceptions [37]. For instance, a study among African migrants in the USA found acculturation to be associated with an increased odds of type 2 diabetes diagnosis [38]. Furthermore, among Arab-speaking immigrants to Australia, a higher degree of acculturation was associated with better glycaemic management [39].

Many migrant populations encounter challenges in accessing healthy food on migration, and the consumption of unhealthy foods has been shown to increase the risk of type 2 diabetes in a dose–response manner [40], as reflected by the high burden of obesity and type 2 diabetes following migration from LMICs to Europe [41, 42]. A recent scoping review found that LMIC migrants living in HICs faced challenges in accessing fresh, traditional, healthier food due to structural and family-level barriers that affected the healthiness of the foods acquired [43]. In the Research on Obesity and Diabetes among African Migrants (RODAM) study, stronger adherence to a ‘mixed’ dietary pattern was associated with a higher 10 year atherosclerotic CVD risk in Ghanaian migrants living in Europe [44]. There is also evidence that a large proportion of migrants do not meet physical activity guidelines [45, 46], which further increases their type 2 diabetes risk [47].

Having consistent routines or everyday practices to follow in managing type 2 diabetes is reported to enhance diabetes management among migrant populations [48, 49]. However, studies report differences in support by gender. For instance, among Hispanic men and women with type 2 diabetes, women were less likely to receive support, faced more barriers, reported less self-efficacy and had lower levels of self-care adherence than men [50, 51]. Although men reported higher levels of support, perceived support was consistently correlated with better self-efficacy in women but not in men [50]. In another study, Pakistani women in Spain encountered more inhibitors than enablers to following a healthy diet [52]. There are large socioeconomic inequalities in CVD outcomes among people with type 2 diabetes, and the effect of education level is particularly strong in women, highlighting the need to take these aspects into account when designing tailored primary prevention management strategies [53].

Social determinants of health (SDOH), which are defined by the WHO as the conditions in which people live, learn, work, play, worship and age and which affect a wide range of health aspects, contribute to the high risk of type 2 diabetes and related outcomes in migrants [54]. SDOH include SES (economy, education, occupation), the food environment, access to healthcare of decent quality, the neighbourhood and physical environment and the social context [54]. Most migrant populations from LMICs living in HICs are congregated at the lower end of the socioeconomic structure and often reside in disadvantaged neighbourhoods in the destination countries [2]. Those with a lower SES are more likely to develop type 2 diabetes, experience more complications and die sooner than those higher up the SES ladder [55–59].

Despite living for decades in recipient countries large proportions of migrants from LMICs experience unemployment, low incomes, lack of education and low health literacy [60]. This limits their opportunities for social integration and access to healthy food options, safe walking environments, social support systems, healthcare providers and quality of care. Their disadvantageous social position poses several challenges in relation to engaging with and maintaining a healthy lifestyle, all of which can escalate type 2 diabetes risk [2, 61]. Life-course exposure based on the length of time living in a resource-deprived environment defined by poverty, lack of quality education or lack of healthcare further significantly impacts disparities in type 2 diabetes risk, diagnosis and outcomes [62–64].

In addition, in the RODAM study, the association of SES with type 2 diabetes risk differed according to context. A higher SES was associated with decreased type 2 diabetes risk among Ghanaian migrants in an urban context in Europe whereas it was associated with increased type 2 diabetes risk in a rural context in Ghana [65]. Furthermore, genetic predisposition and epigenetic changes on migration can influence insulin secretion and action and subsequently type 2 diabetes risk. In an epigenome-wide association study of people of Ghanaian origin, lifestyle factors in combination affect DNA methylation, influencing the risk of type 2 diabetes in adults living in different contexts including Europe [66].

Although SDOH represent barriers to health equity and contribute to unfair and avoidable differences in health status within and between countries [54], the integration of SDOH into clinical trials has been limited [67]. Language barriers, economics and logistical challenges (e.g. transport costs, lack of childcare or difficulty in getting time off work) often prevent marginalised groups from being informed about and/or enrolled in clinical trials, which also impacts the quality of studies and limits the generalisability of the results [68].

## Prevention and management of type 2 diabetes in migrants from LMICs living in HICs

The susceptibility to type 2 diabetes among migrant populations calls for a concerted effort to implement effective prevention and management strategies for at-risk groups. Identification of structural and sociocultural barriers to and facilitators of lifestyle modification is crucial for successful health promotion in migrant populations at high risk for type 2 diabetes [69]. Existing consensus guidelines emphasise weight management through dietary change and physical activity [70], an approach that has been shown to be effective in intervention trials of diabetes prevention programmes in many populations across the world [71–74]. For instance, in the US Diabetes Prevention Program, the effects of intense lifestyle interventions were shown across all ethnic groups [74]. Furthermore, an individual participant data meta-analysis found that lifestyle interventions in high-risk South Asian populations (four from Europe and two from India) resulted in a clinically relevant 35% relative reduction in diabetes incidence, consistent across subgroups, despite modest changes in adiposity [75, 76].

### Barriers to and facilitators of lifestyle changes

Although there have been some successes in tackling type 2 diabetes in migrant populations, there are still many issues to be considered. Structural, cultural, social and religious beliefs, low levels of health literacy and barriers related to gender and language aspects are some of the factors that impact self-management among migrants with type 2 diabetes living in HICs [77–79].

Cultural beliefs can significantly influence both preventive and curative healthcare-seeking behaviours as well as adherence to treatment advice [80]. A systematic review examining the evidence on culturally competent interventions tailored to the needs of people with diabetes from minority ethnic groups concluded that structured interventions, tailored by integrating elements of culture, language, religion and health literacy skills, produced a positive impact on a range of important patient outcomes [81]. Several trials targeting primary or secondary prevention of type 2 diabetes in high-risk migrant populations have shown beneficial results in terms of reductions in BMI, HbA_1c_ and lipid levels and less severe insulin resistance when cultural barriers including health beliefs and cultural practices are addressed [61, 69, 82–90]. Another systematic review evaluating the effectiveness of tailoring community-based diabetes interventions to Asian migrant cultures revealed that culturally tailored programmes were effective in improving individuals’ objectively measured clinical outcomes, in particular HbA_1c_ levels, and psycho-behavioural outcomes [91]. Asian migrants with type 2 diabetes were also highly satisfied with bilingual healthcare providers and bilingual educational programmes [91].

A systematic review of pragmatic community-based interventions among South Asian populations found that they had promise regarding weight loss and reductions in blood pressure and lipid levels but there was limited evidence for effects on behavioural change [92]. The inclusion of individual feedback and support from community workers in socioeconomically vulnerable areas seemed important in the recruitment process and the authors concluded that further development of culturally tailored trials is required [92].

The first 5 years after migration may provide a window of opportunity to provide targeted interventions to ensure the maintenance of healthy dietary habits [93]. Barriers to optimal management of diabetes among migrants include dietary struggles related to unfamiliar diets recommended by healthcare professionals for managing diabetes, difficulties with cooking healthily, cultural influences on dietary habits and long working hours due to the high cost of living [48, 94, 95]. Dutch and Swedish studies have concluded that national dietary care is not tailored to the needs of people at high risk and should consider migrants’ expectations and cultural differences in dietary habits and address the role of the whole family [69, 96]. Thus, more emphasis is needed on providing culturally relevant nutritional interventions [97].

Facilitators of lifestyle modification and diabetes management may be connected to the presence of social support and family support [69, 98, 99], with an important barrier to making dietary change being lack of self-efficacy [96]. Interventions targeting self-efficacy and illness perceptions, focusing on building self-confidence and how people perceive their diabetes, may improve self-care activities and reduce diabetes distress among migrants with type 2 diabetes [100]. Further, culturally sensitive diabetes self-management programmes have the potential to enhance health literacy [83]. A study of Somali women in Norway concluded that health literacy, including poor access to health information and tailored physical activity services, may impact lifestyle in this group at high risk for type 2 diabetes [101].

## Measures to improve the prevention and management of type 2 diabetes in migrants from LMICs living in HICs

### Culturally sensitive interventions in the prevention and management of type 2 diabetes

Type 2 diabetes is a growing public health concern, both in general and in migrants from LMICs more specifically, who often face social, economic and cultural challenges that increase their risk of developing this condition. Preventive, culturally sensitive strategies that target language barriers, health literacy, cultural differences in lifestyle, access to healthcare and socioeconomic inequalities are crucial. Huge gains can be achieved by eliminating structural and individual barriers that increase key risk factors for type 2 diabetes and management, especially non-adherence to lifestyle and treatment recommendations. More emphasis should be placed on awareness creation and identification of facilitators to improve adoption of a healthy lifestyle and patient education by trusted individuals.

**Fig. 3:**
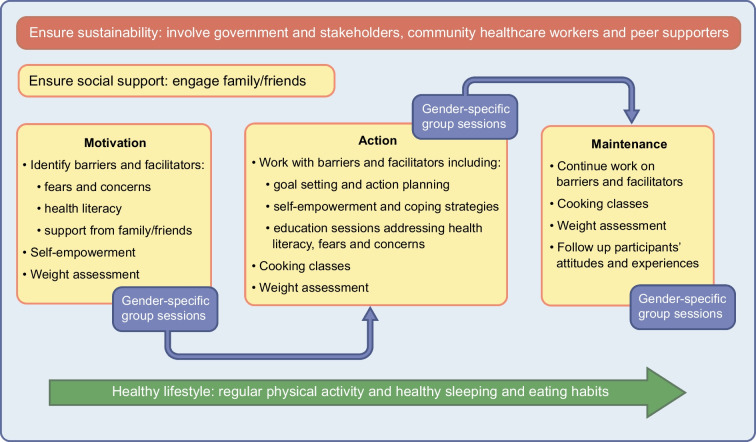
Illustration of a culturally sensitive lifestyle intervention addressing SDOH that includes gender-specific group sessions and focuses on structural and individual barriers. This figure is available as part of a downloadable slideset

There is strong evidence from several countries that culturally sensitive interventions with tailoring of cardiovascular risk strategies that target SDOH, health literacy, self-empowerment, acculturation, family involvement, beliefs, cultural barriers and facilitators by gender [21, 69, 76, 85, 86, 102, 103] may be warranted for the prevention and management of type 2 diabetes in migrants from LMICs (Fig. 3).

Several culturally adapted RCTs have shown successful results with beneficial effects on cardiometabolic risk (Table 1) [75, 86, 87, 91, 104–107]. However, stakeholders and governments seldom implement or scale up evidence-based findings into healthcare. Although some initiatives may be carried out by enthusiasts, implementing new intervention strategies in healthcare systems requires leadership and support from governments, and stakeholders are needed for sustainable implementation. Otherwise, healthcare providers will continue to apply ‘treatment as usual’, perpetuating existing health inequalities. Culturally/individually adapted prevention is not only medically warranted but also often requested by people at risk [108].

**Table 1:** Culturally adapted RCTs with statistically significant effects on cardiometabolic risk in migrants from LMICs living in HICs

Study and setting	Population	Study design	Intervention	Intervention duration	Control	Outcome	Statistically significant effects
Jenum et al [75] Europe and India	South Asian adults (*n*=6 studies; *N*=1816)	Meta-analysis of available RCTs 2018	Lifestyle modification incorporating diet and physical activity	5 months to 3 years	UC	Risk reduction in T2D incidence	Absolute risk reduction: 7.4% Relative risk reduction: 35%
Joo [91] Asian immigrant cultures	South Asian migrants with T2D aged >50 years (*n*=9 studies; *N*=514)	Systematic review and meta-analysis of RCTs or quasi experimental designs published since 2000	Culturally tailored programme in community-based setting	6 weeks to 12 months	UC	Improvement in HbA_1c_ (*n*=6 studies) Psycho-behavioural outcomes: knowledge of diabetes, self-efficacy, DQOL (*n*=4–7 studies) Satisfaction (*n*=8 studies)	HbA_1c_ improved in 5/6 studies Diabetes knowledge increased in 3/7 studies Self-efficacy increased in 3/5 studies DQOL improved in 4/4 studies All studies reported moderate to high participant satisfaction with the programmes
Pekmezaris et al [104] USA	Latino adults with T2D (*N*=240)	RCT	Diabetes telehealth management (DTM) + comprehensive outpatient monitoring (COM)	6 months	COM	Change in HbA_1c_, SBP and DBP	HbA_1c_: DTM+COM −0.7%; COM −0.2% SBP: DTM+COM −8.7 mmHg; COM +2.4 mmHg DBP: DTM+COM −3.2 mmHg; COM + 1.0 mmHg
Rosas et al [106] USA	Latino adults at risk of T2D (*N*=207; BMI 30–60 kg/m^2^)	RCT	Case management (CM) intervention with and without community healthcare workers (CHWs)	24 months	UC	Change in weight at 6, 12 and 24 months	Compared with UC at 6 months: CM+CHW −2%; CM −1.6% Compared with UC at 12 and 24 months: NS
Rosas et al [105] USA	Latino adults at risk of T2D (*N*=191; BMI >24 kg/m^2^)	RCT	‘Vida Sana’: family-based orientation session and 22 group sessions over 12 months	24 months	UC	Change in weight at 12 and 24 months	Compared with UC at 12 months: −2.1 kg (−5%) Compared with UC at 24 months: NS
Siddiqui et al [86, 107] Sweden	Adult Middle Eastern migrants at risk of T2D (*N*=64; BMI >28 kg/m^2^ and/or insulin resistance)	RCT	Weekly, gender-specific group sessions addressing lifestyle change	4 months	UC	Change in weight, BMI, LDL, ISI Mental well-being evaluated as the OR of scoring lower at follow-up on MADRS-S and HAD	Compared with UC: Weight: −0.4%/month; BMI: −0.4%/month; LDL: −2.1%/month; ISI: +10.9%/month MADRS-S: OR 5.9%; HAD: OR 4.4%
Yeh et al [87] USA	Chinese adults with prediabetes (*N*=60)	RCT	DPP lifestyle intervention: 12 biweekly core sessions and 6 monthly post-core sessions covering physical activity, healthy eating, stress reduction and problem-solving	12 months	Quarterly mailing of diabetes prevention information	Change in weight at 6 and 12 months	Weight loss at 6 months: DPP −3.5%; control −0.1% Weight loss at 12 months: DPP −3.3%; control: −0.3%

CHW, community healthcare worker; CM, case management intervention; COM, comprehensive outpatient monitoring; DBP, diastolic BP; DPP, Diabetes Prevention Program; DQOL, diabetes-related quality of life; DTM, diabetes telehealth management; HAD, Hospital Anxiety and Depression scale; ISI, Insulin Sensitivity Index; MADRS-S, Montgomery Åsberg Depression Rating Scale – self report; NS, non-significant; SBP, systolic BP; T2D, type 2 diabetes; UC, usual care

Salient cultural values emphasised by individuals at risk of type 2 diabetes and stakeholders underscore the importance of family and community support for behavioural change [109]. Involving at-risk individuals and family members in the cultural adaptation of prevention programmes contributes to congruence with the cultural values of the study populations and further strengthens the cultural relevance of the interventions [109]. Furthermore, community engagement approaches for the recruitment of minority ethnic groups can improve the integration of migrant populations, for instance engaging diverse community stakeholders in the recruitment process, often through study-specific or long-term community advisory boards. However, there is a lack of comparative studies on the effectiveness of different levels of community engagement for different migrant groups [110]. In a qualitative study of minority ethnic populations with type 2 diabetes, participants described satisfaction with self-management support delivered by community healthcare workers and peer supporters and the open and trusting relationships that developed, in contrast to support delivered by and relationships with clinical providers [111]. Furthermore, they described how they benefitted from the social support of fellow participants, community healthcare workers and peer supporters and that they changed a range of health-related behaviours and had more confidence in handling their own condition and interacting with clinicians [111].

### Translation of evidence into policy and practice

A recent review reinforced the importance of policymakers involving the whole community of minority ethnic and migrant groups in lifestyle intervention programmes [76]. A study that aimed to identify the best strategy to reach individuals at high risk of type 2 diabetes and CVD in a heterogeneous population showed that the choice of communication channels is important [112]. When addressing migrants from LMICs, directing approaches to the community rather than to individuals may be more effective [112].

There is also a need to empower migrant communities to discuss type 2 diabetes in a manner that prevents stigmatisation of the condition. Four in five adults with diabetes experience diabetes-related stigma and one in five experience discrimination [113]. Information on type 2 diabetes‐related risk factors, potential side effects of treatment and benefits of treatment needs to be provided in a way that is easily understandable, taking into account cultural factors related to language barriers, lifestyle (e.g. physical activity levels, cultural foods) and socioeconomic background. In addition, regular follow‐up is necessary to confirm if the advice provided has been understood and is being successfully followed.

It is vital for healthcare services to promote multicultural workforces, especially in areas with a high concentration of migrants, and provide a migrant-friendly atmosphere to encourage the uptake of services by migrant populations. There is also a need to train healthcare employees, especially support staff, about culturally sensitive care, which refers to the ability to provide equitable and high-quality care to all individuals, regardless of ethnic background, language proficiency or culture, as they play a crucial role in the care of people with type 2 diabetes [114]. Healthcare professionals should reflect on the populations they serve, recognising and acknowledging the significance of culture in migrants’ health beliefs and behaviours, and healthcare provision should be tailored to cater to the culturally specific requirements of migrant groups.

### Research gaps

The following research gaps should be addressed:

- Future research should address SDOH and focus on gender roles, norms and inequalities in high-risk migrant groups in different settings/countries to examine their health beliefs and cultural practices. These findings should be used to develop best practice guidelines and should be incorporated into culturally tailored interventions [84]. Future studies should further explore how health literacy can improve type 2 diabetes prevention and management in migrant populations [83], taking gender aspects into account.
- Despite the long-standing evidence for SDOH as key factors in both diabetes risk and outcomes, there is a lack of systematic investigation in the existing scientific literature [23, 61]. Future research should therefore evaluate the impact of changes in SDOH on diabetes risk and outcomes among migrant populations.
- A recent review found that social support, including peer support and self-management education, significantly improved diabetes management in minority populations in the USA and UK. However, more studies globally among migrants from LMICs living in HICs are needed to address this evidence gap [115].
- The limited understanding of the mechanisms underlying the high burden of type 2 diabetes among migrants from LMICs living in HICs calls for longitudinal multinational research using real-world data from these populations and a gender lens to shed light on the key factors involved.
- Larger studies testing the effectiveness of long-term culturally sensitive interventions on hard endpoints, including major adverse cardiovascular events, are still lacking and there is an urgent need to address this gap in knowledge among migrant populations.

## Conclusions

The prevention and management of type 2 diabetes in migrants from LMICs living in HICs calls for substantial future investments to tackle the burden and close the health inequality gaps (see Text box: Summary of the prevention of type 2 diabetes in migrant populations from LMICs).


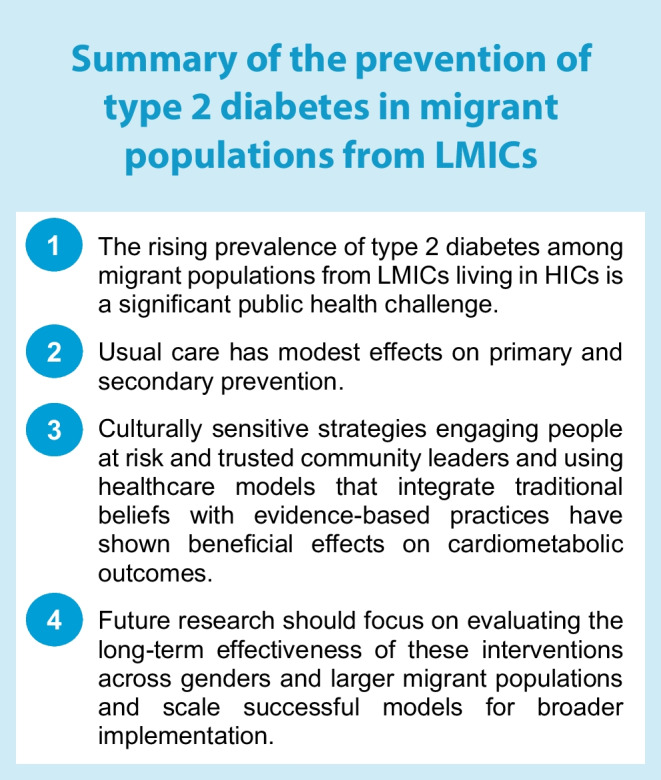


The disproportionately high burden of type 2 diabetes among migrant populations is costly in terms of economics and human impact. Understanding the biological, pre- and post-migration‐related and cultural and environmental factors that drive the high prevalence of type 2 diabetes and related complications, low uptake of preventive measures and poor glycaemic management in migrant populations is pivotal to mitigating these inequalities.

Future preventive actions should target structural and individual barriers to and facilitators of lifestyle change in migrants from LMICs living in HICs through culturally sensitive interventions addressing behaviour change. To ensure sustainability, policymakers should be involved, migrant communities and multicultural workforces should be empowered and healthcare providers should be educated in culturally sensitive interventions.

Future research should evaluate how SDOH, health beliefs and health literacy impact type 2 diabetes prevention and management and investigate the impact of long-term culturally sensitive interventions on major adverse cardiac events and all-cause and cause-specific mortality in migrants with type 2 diabetes from LMICs.

## Supplementary Information

Below is the link to the electronic supplementary material.

Slideset of figures (PPTX 1.37 MB)

Supplementary file2 (PDF 88 KB)

## Abbreviations

HICs: High-income countries
LMICs: Low- and middle-income countries
RODAM: Research on Obesity and Diabetes among African Migrants
SDOH: Social determinants of health
SES: Socioeconomic status
