# Supplementary material for: Prevention of type 2 diabetes in migrant populations from low- and middle-income countries living in high-income countries
Source: Diabetologia. 2025 Jun 7;68(11):2405–19. doi: 10.1007/s00125-025-06465-9 (PMC12534338; doi:10.1007/s00125-025-06465-9)
Supplement: Supplementary file 1 — Slideset of figures (PPTX 1.37 MB) [file 125_2025_6465_MOESM1_ESM.pptx]

## Slide 1
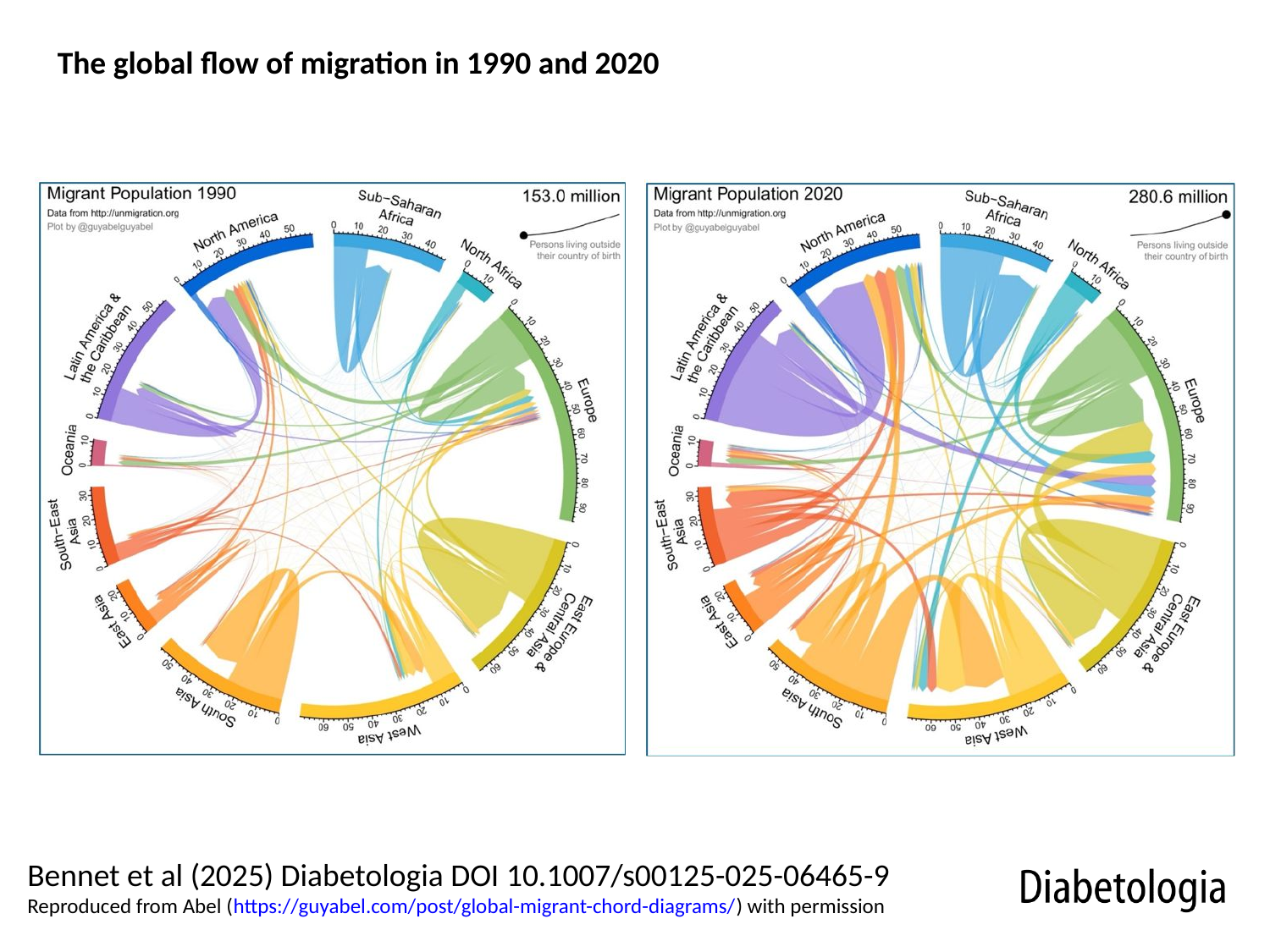

The global flow of migration in 1990 and 2020
Bennet et al (2025) Diabetologia DOI 10.1007/s00125-025-06465-9
Reproduced from Abel (https://guyabel.com/post/global-migrant-chord-diagrams/) with permission

## Slide 2
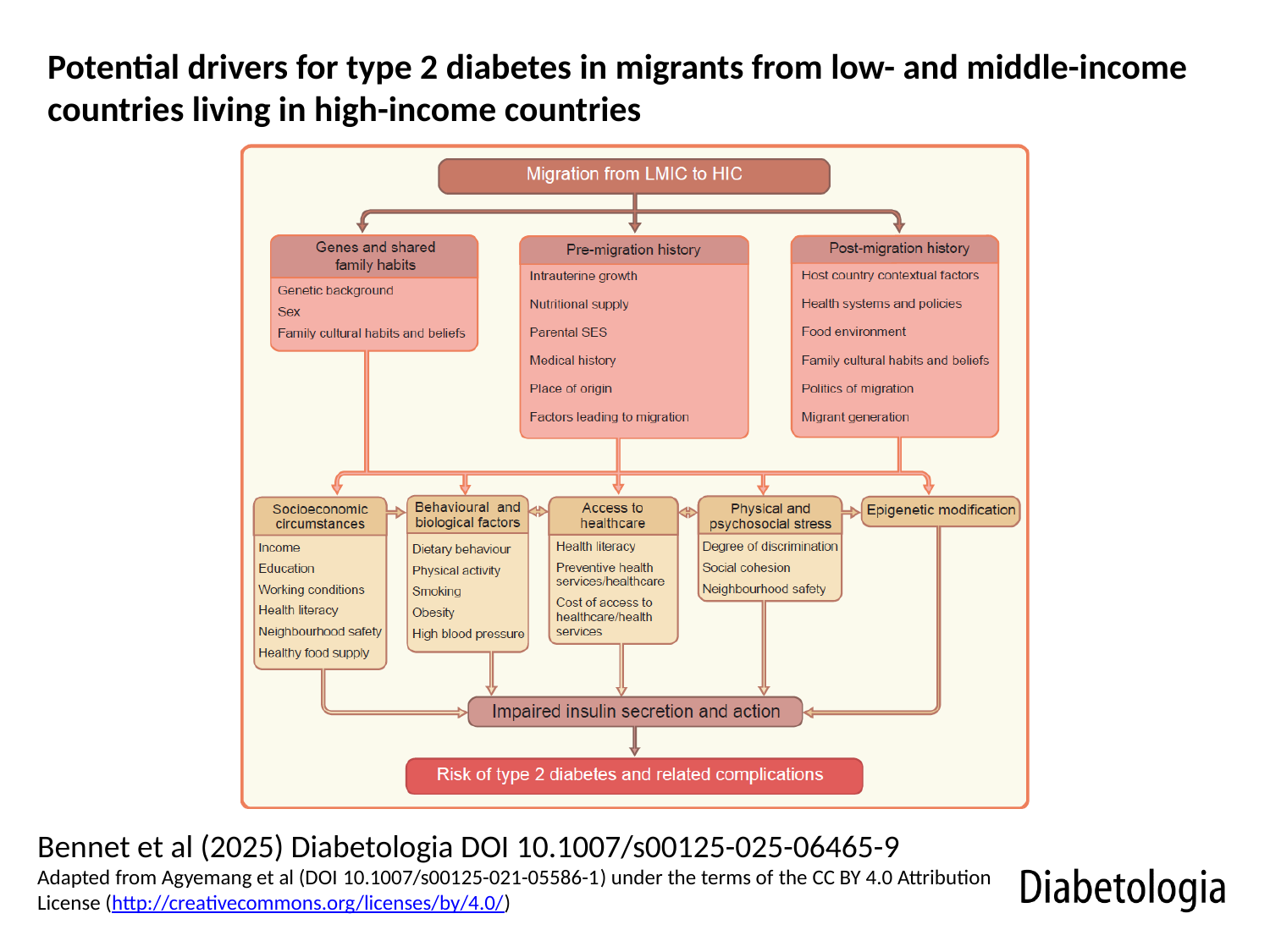

Potential drivers for type 2 diabetes in migrants from low- and middle-income countries living in high-income countries
Bennet et al (2025) Diabetologia DOI 10.1007/s00125-025-06465-9
Adapted from Agyemang et al (DOI 10.1007/s00125-021-05586-1) under the terms of the CC BY 4.0 Attribution License (http://creativecommons.org/licenses/by/4.0/)

## Slide 3
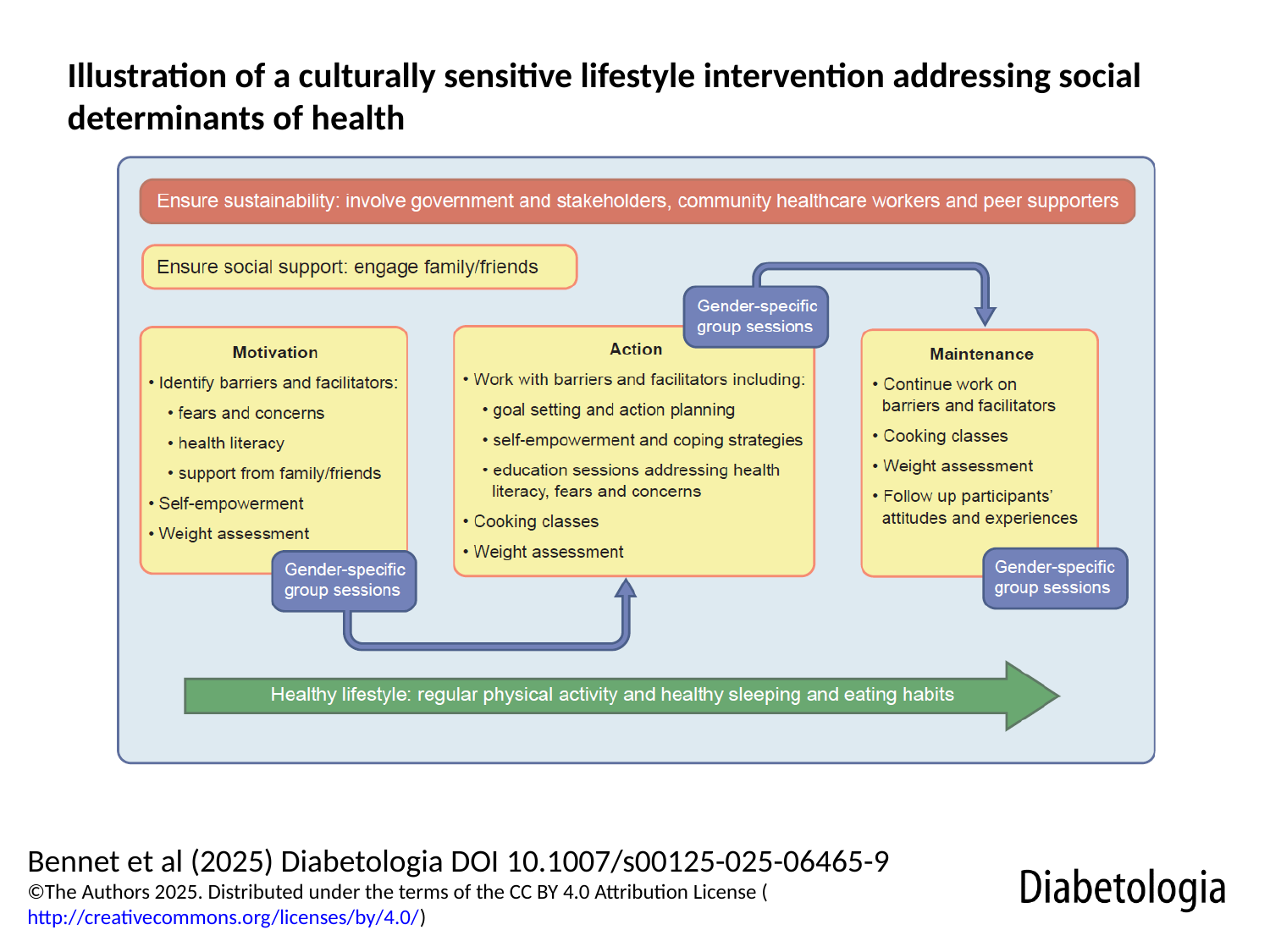

Illustration of a culturally sensitive lifestyle intervention addressing social determinants of health
Bennet et al (2025) Diabetologia DOI 10.1007/s00125-025-06465-9
©The Authors 2025. Distributed under the terms of the CC BY 4.0 Attribution License (http://creativecommons.org/licenses/by/4.0/)
