# Supplementary material for: Prevention of type 2 diabetes in migrant populations from low- and middle-income countries living in high-income countries
Source: Diabetologia. 2025 Jun 7;68(11):2405–19. doi: 10.1007/s00125-025-06465-9 (PMC12534338; doi:10.1007/s00125-025-06465-9)
Supplement: Supplementary file 2 — Supplementary file2 (PDF 88 KB) [file 125_2025_6465_MOESM2_ESM.pdf]

Electronic supplementary material (ESM)

ESM Fig. 1. Animated chord diagram of the global flow of migration, 1990–2020 (https://guyabel.com/post/global-migrant-chord-diagrams/area-time-abel.mp4). The chords in the diagram represent the connections between the places of birth (at the base of the chords) and places of residence (at the arrow heads of the chords). The width of the base of a chord corresponds to the size of the migrant population in millions. Chords are ordered relative to their size, with the largest migrant populations plotted at the beginning of the region segments. The ordering of chords jumps around over time as the relative rankings of the largest foreign-born populations change in each region. Reproduced from with permission. (You may need to right click, select 'show controls' and press play to start the animation depending on your browser).
